# Mechanisms of B Cell Receptor Activation and Responses to B Cell Receptor Inhibitors in B Cell Malignancies

**DOI:** 10.3390/cancers12061396

**Published:** 2020-05-28

**Authors:** Dimitar G. Efremov, Sven Turkalj, Luca Laurenti

**Affiliations:** 1 Molecular Hematology, International Centre for Genetic Engineering and Biotechnology, 34149 Trieste, Italy; SVEN.TURKALJ@studenti.units.it; 2 Department of Hematology, Fondazione Policlinico Universitario A Gemelli IRCCS, 00168 Rome, Italy

**Keywords:** chronic lymphocytic leukemia, lymphoma, B-cell receptor, BTK, PI3K, SYK

## Abstract

The B cell receptor (BCR) pathway has been identified as a potential therapeutic target in a number of common B cell malignancies, including chronic lymphocytic leukemia, diffuse large B cell lymphoma, Burkitt lymphoma, follicular lymphoma, mantle cell lymphoma, marginal zone B cell lymphoma, and Waldenstrom’s macroglobulinemia. This finding has resulted in the development of numerous drugs that target this pathway, including various inhibitors of the kinases BTK, PI3K, and SYK. Several of these drugs have been approved in recent years for clinical use, resulting in a profound change in the way these diseases are currently being treated. However, the response rates and durability of responses vary largely across the different disease entities, suggesting a different proportion of patients with an activated BCR pathway and different mechanisms of BCR pathway activation. Indeed, several antigen-dependent and antigen-independent mechanisms have recently been described and shown to result in the activation of distinct downstream signaling pathways. The purpose of this review is to provide an overview of the mechanisms responsible for the activation of the BCR pathway in different B cell malignancies and to correlate these mechanisms with clinical responses to treatment with BCR inhibitors.

## 1. Introduction

The B cell receptor (BCR) is a transmembrane signaling complex that is expressed by most normal and malignant B lymphocytes and plays a key role in regulating the growth, differentiation, and function of these cells. It is composed of a membrane immunoglobulin molecule, which functions as the antigen recognition unit, and a heterodimer of the proteins CD79A and CD79B, which functions as the signaling unit. Binding of the membrane immunoglobulin to antigen generates a signal that is transduced by a complex network of various kinases, phosphatases, adaptor proteins, and transcription factors. This signal can induce a variety of cellular responses, including proliferation, differentiation, adhesion, survival, anergy, or apoptosis. The outcome is determined by the relative activity of the various downstream signaling molecules that transduce the BCR signal. In addition to antigen binding, antigen-independent mechanisms have been shown to activate the BCR pathway in certain B cell malignancies and to contribute to the expansion and survival of the malignant B cells.

In this review, we outline the mechanisms responsible for the chronic activation of the BCR pathway in various B cell malignancies and describe how these different mechanisms affect the signaling pathways and cellular responses that are regulated by the BCR signal. In addition, we summarize clinical experiences with different BCR inhibitors and correlate the clinical responses with mechanisms of BCR pathway activation. 

## 2. BCR Signaling in Normal and Malignant B Cells

**Figure 1:**
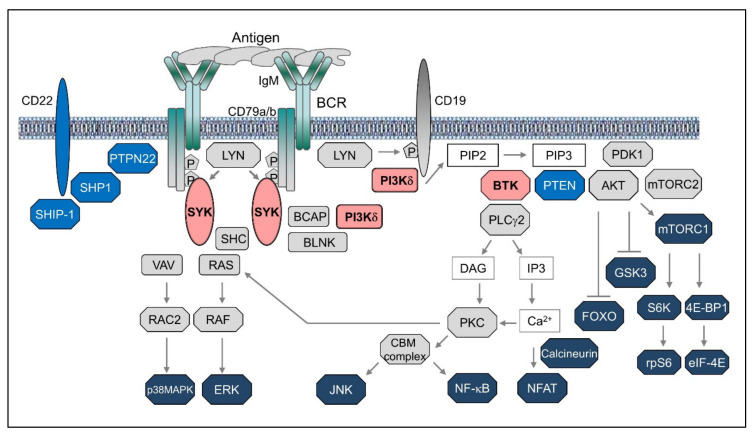
Overview of B-cell receptor signaling pathways.

The initial events resulting in activation of the BCR have still not been fully elucidated, but most available evidence suggests that antigen binding induces a reorganization of the actin cytoskeleton leading to local convergence of monomeric or oligomeric BCR units and their assembly into signaling microclusters [1,2,3]. These clusters then recruit members of the SRC-family of kinases (SFKs), such as LYN, FYN, or BLK, that phosphorylate the tyrosine residues within the immunoreceptor tyrosine-based activation motifs (ITAMs) of CD79A and CD79B (Figure 1). The phosphorylated ITAMs then serve as binding sites for the kinase SYK, which becomes activated through a multistep process that involves phosphorylation by SRC family kinases and trans-autophosphorylation [4]. Once activated, SYK propagates the BCR signal by phosphorylating the adaptor proteins BLNK, BCAP, and SHC, which then serve as a scaffold for the recruitment of other signaling molecules that together form a large multimolecular complex defined as the BCR signalosome [5]. The BCR signal is further propagated by the lipid kinase PI3Kδ, which is recruited to the signalosome by binding to BCAP or CD19 and becomes activated through a conformational change in the regulatory p85 subunit that exposes the catalytic p110δ subunit [6]. Activated PI3Kδ then phosphorylates the membrane phospholipid phosphatidylinositol-4,5-bisphosphate (PIP2) and converts it into phosphatidylinositol-3,4,5-triphosphate (PIP3), which then recruits several important downstream signaling molecules and therapeutic targets. One of these is the kinase BTK, which is subsequently activated by phosphorylation by SRC family kinases and autophosphorylation. BTK then phosphorylates and activates PLCγ2, which hydrolyses PIP2 to generate inositol trisphosphate (IP3) and diacylglycerol (DAG). Binding of IP3 to its receptor, a calcium channel located in the endoplasmic reticulum, results in release of calcium into the cytosol and activation of the phosphatase calcineurin, which then dephosphorylates and activates the transcription factor NFAT [7]. In addition, calcium together with DAG activate protein kinase C (PKC), which phosphorylates the adaptor protein CARD11 and induces the formation of a CARD11–BCL10–MALT1 (CBM) signaling complex that activates the transcription factor NF-κB [8]. 

Another key signaling molecule that is recruited to the cellular membrane by PIP3 is the serine/threonine kinase AKT, which becomes activated following phosphorylation by PDK1 and the mTORC2 complex [9]. AKT has numerous substrates that play important roles in regulating cell growth and survival. Among these are the FoxO transcription factors and the kinase GSK3, which are both inactivated by AKT-mediated phosphorylation. Inactivation of FoxO transcription factors results in reduced expression of certain proapoptotic and cell-cycle inhibitory proteins, whereas inactivation of GSK3 inhibits the turnover and prolongs the half-life of several proteins that positively regulate cell survival and proliferation. In addition, AKT phosphorylates and activates the mTORC1 complex, which then increases the rate of protein translation by activating the ribosomal protein S6 kinase and the eukaryotic initiation factor 4E (eIF4E).

Other signaling molecules that are activated downstream of the BCR are the mitogen-activated protein kinases ERK, JNK and p38MAPK [10]. These kinases regulate a number of transcription factors, including Elk1, c-Myc, c-Jun, ATF2, and Max, which are important for B cell proliferation and survival.

The BCR signal is negatively regulated by various inhibitory receptors, such as CD22, CD72, FCγRIIB, and SIGLEC10, and phosphatases, such as SHP1, PTPN22, SHIP1, and PTEN [11,12]. SHP1 and PTPN22 terminate the BCR signal by dephosphorylating certain BCR-proximal signaling components, including CD79A, CD79B, SFKs, SYK, and BLNK, whereas PTEN and SHIP1 function by dephosphorylating PIP3. Expression of these negative regulators can vary across different B cell malignancies and can affect the capacity of the malignant cells to transduce the BCR signal. For example, SIGLEC10 is often downregulated in human B-cell lymphoma cell lines compared to normal B cells and knockdown of this receptor in a murine model has been shown to result in enhanced BCR signaling [13,14]. Similarly, downregulation of PTEN has been reported in 55% of germinal center B-cell-like (GCB) diffuse large B cell lymphomas (DLBCLs) and results in constitutive activation of the PI3K/AKT pathway [15]. Downregulation of SHP1 has also been observed across several B cell malignancies and can result in antigen-independent activation of certain downstream BCR signaling pathways (described in greater detail later). Together, these data suggest that perturbations of the BCR signaling machinery are frequent in B cell malignancies and likely to contribute to the pathogenesis of these disorders.

The complexity of the BCR signaling network is further increased by the existence of parallel pathways of activation and the substantial crosstalk between the different downstream signaling molecules. These features are sometimes cell type specific and may account in part for the different activity of the various BCR inhibitors in different B cell malignancies. For example, in addition to PI3Kδ, the related kinase PI3Kα has been implicated in signaling through the BCR in Activated B cell-like (ABC) DLBCL [16,17], providing a potential explanation for the greater clinical activity of the dual PI3Kα/δ inhibitor copanlisib compared to the PI3Kδ inhibitor idelalisib in patients with DLBCL [18,19]. Similarly, a recent study from our group showed that two pathways downstream of the BCR are involved in inactivation of the kinase GSK3 in chronic lymphocytic leukemia (CLL) cells, resulting in a different capacity of SYK, BTK, and PI3Kδ inhibitors to reduce the expression of the antiapoptotic protein Mcl-1, which is negatively regulated by GSK3 [20]. Finally, although BTK is located downstream of PI3K, several studies have suggested that there is also crosstalk in the opposite direction and that BTK can enhance the activity of the PI3K/AKT pathway in BCR-stimulated cells [21,22,23,24,25,26]. The mechanism behind this effect is still not completely understood, but involvement of BTK in the phosphorylation of the adaptor BCAP and in the production of the PI3K substrate PIP2 have been proposed as potential explanations [22,24]. This crosstalk could also provide an explanation for the partial inhibition of AKT following treatment of CLL cells and lymphoma cell lines with the BTK inhibitor ibrutinib in vitro and in vivo [27,28,29,30]. 

**Figure 2:**
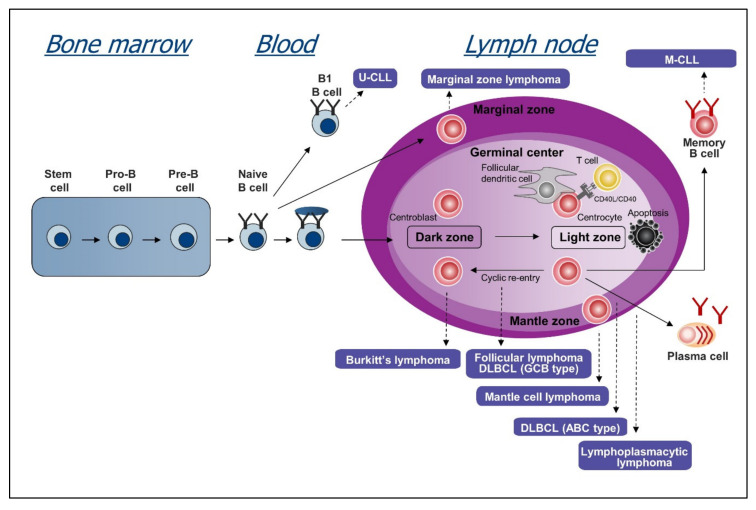
B cell development and cell of origin of B cell malignancies covered in this review.

Another possible explanation for the variable activity of BCR inhibitors in different B cell malignancies is the mechanism of BCR activation. Studies that were conducted over the last 15–20 years have provided compelling evidence that the BCR pathway is activated in CLL, DLBCL, Burkitt lymphoma (BL), follicular lymphoma (FL), mantle cell lymphoma (MCL), marginal zone lymphoma (MZL), and Waldenstrom’s macroglobulinemia (WM) (Figure 2). However, these studies also revealed important differences in the proportion of cases with an activated BCR pathway and the mechanism of BCR pathway activation. The following sections will describe our current understanding of these mechanisms in the different disease entities and how these relate to responses to treatment.

## 3. BCR Signaling in Chronic Lymphocytic Leukemia

CLL is characterized by the expansion of a subset of mature B lymphocytes that express the surface markers CD5, CD19, and CD22 and low levels of surface IgM and CD79B. Substantial evidence suggests a central role of BCR signaling in disease development and progression. Seminal studies on immunoglobulin gene structure uncovered two main subsets of CLL: unmutated CLL (U-CLL) harboring 98% or more homology to the germline immunoglobulin heavy chain variable (IGHV) gene sequences and mutated CLL (M-CLL), where less than 98% IGHV gene homology to the germline is observed [31]. The fact that U-CLL patients have significantly worse prognosis than M-CLL patients hinted that BCR structure is a key predictor of disease progression [32,33]. 

Apart from these characteristics related to the degree of somatic hypermutation (SHM), CLL BCRs also show a peculiar pattern of IGHV and immunoglobulin light chain variable (IGLV) gene usage which is dominated by the presence of particular IGHV and IGLV gene combinations. BCRs encoded by these IGHV/IGLV combinations and having particular HCDR3 structures have been named stereotyped BCRs and have been identified in approximately one-third of CLL cases, whereas they are rarely seen in normal B lymphocytes [34,35,36,37]. Considering that the likelihood of this happening by chance is virtually negligible, the occurrence of stereotyped BCRs has been taken as evidence that CLL cells are selected because of particular antigen-binding properties. Based on IGHV/IGLV association and HCDR3 properties, the stereotyped receptors have been divided into “stereotyped subsets”.

Soluble immunoglobulins derived from CLL cells have been reported to display shared reactivity towards self-antigens, commonly more than one [38,39,40,41,42,43]. Such polyreactivity is primarily observed with U-CLL immunoglobulins and includes binding to IgG, ssDNA, dsDNA, vimentin, filamin A, cofilin-1, phosphorylcholine on oxidized LDL, cardiolipin, nonmuscle myosin heavy chain IIA, and stromal cell antigens. Importantly, many of the abovementioned self-antigens are found on apoptotic blebs [43], hinting that microenvironmental apoptosis is an important disease driver. Additionally, reactivity with foreign antigens has been reported in some cases, including *Streptococcus pneumoniae* polysaccharides [40], β-(1,6)-glucan on filamentous fungi [44], cytomegalovirus phosphoprotein pUL32 [45], HIV-1 envelope gp41, influenza hemagglutinin, and hepatitis C virus E2 protein [46]. 

Reactivity with any of these antigens could account for the chronic activation of the BCR pathway that is frequently observed by gene expression or phospho-protein profiling analysis of CLL cells. Such evidence is particularly seen in CLL cells isolated from lymph nodes, which typically display high levels of BCR and NF-κB target genes [47] and express constitutively activated BCR signaling molecules, including LYN [48], SYK [49], PI3K [50], BTK [29], PKCβ [51], ERK [52], NF-κB [53], and NFAT [52]. Importantly, enhanced activation of these molecules correlates with inhibition of spontaneous apoptosis, suggesting a pro-survival role for BCR signals [29,48,49,50]. Indeed, the BCR-induced constitutive SYK activation has been shown to upregulate the antiapoptotic protein Mcl-1 [49] by activating the PI3K/AKT pathway [54,55]. Notably, prolonged AKT activity results in increased mTORC1 and reduced GSK3 activity, with a resulting increase in Mcl-1 protein translation and inhibition of MCL1 degradation, respectively [54,56,57]. 

Further pointing to an important role for the BCR pathway in the pathogenesis of CLL is the fact that a number of signaling molecules that are involved in BCR signal transduction are aberrantly expressed by the leukemic cells. The ZAP-70 protein kinase, which is a SYK homologue that plays a key role in transducing signals through the T cell receptor, is aberrantly expressed mostly in U-CLL patients [58]. Importantly, ZAP-70 associates with CD79B, enhancing BCR signaling and acting as a negative prognostic factor [59]. Interestingly, although ZAP-70 is inefficiently phosphorylated following BCR stimulation, its role in recruiting downstream BCR molecules is preserved [60], hinting that it could interfere with BCR negative regulation rather than being a direct activator. Defective negative regulation is a frequent phenomenon in oncogenic signaling; accordingly, absent or substantially reduced expression of the AKT and ERK negative regulator PHLPP1 is observed in CLL cells, causing an enhanced BCR-mediated AKT, ERK, and GSK3 phosphorylation [61]. An additional mechanism accounting for aberrant AKT activation in CLL consists in the overexpression of the phosphatase PTPN22 [62]. PTPN22 quells LYN activity, thus blunting LYN-mediated activation of a negative regulatory loop involving the inhibitory receptor CD22 and the phosphatase SHIP, which by dephosphorylating PIP3 blocks AKT membrane recruitment and activation. Given that LYN is a major activator of SYK, PTPN22 overexpression also downregulates proximal BCR signaling, including PLCγ2 and MAPK cascade activation. The latter effects may seem counterintuitive given the pro-oncogenic role of the BCR. However, hyperactivation of BCR signalling above a maximum threshold can induce apoptosis in B cells, including CLL cells [63,64]. Thus, PTPN22 overexpression may serve to selectively uncouple AKT from downstream proapoptotic BCR pathways and thus protect CLL cells from tolerance mechanisms that eliminate autoreactive B cells. 

Another AKT regulator, TCL1, is also often overexpressed in CLL cells, especially in the U-CLL subset [65]. TCL1 is a lymphoid oncogene which associates with AKT and ZAP-70 in the proximity of the membrane. More precisely, BCR activation induces and stabilizes AKT-TCL1 complexes on the membrane, potentiating AKT-mediated signals [66]. Importantly, TCL1 is a potent negative prognostic marker in CLL. Consistently, Eµ-TCL1-transgenic mice display an emergence of clonal CD5+/IgM+ B cell expansions resembling IGVH-unmutated human CLL, thus defining TCL1 as a strong CLL oncogene [67,68]. Collectively, these studies indicate that recurrent alterations in the levels of positive and negative BCR signaling regulators intrinsically affect the nature of BCR signaling and may contribute to the pathogenesis of CLL. 

A major step forward in understanding how BCR signals are generated in CLL cells came from the study of Dühren-von Minden and colleagues, who identified cell-autonomous signaling consequent to BCR recognition of internal immunoglobulin epitopes as a novel mechanism of BCR pathway activation in CLL [69]. In this paradigm-shifting study, Dühren-von Minden and colleagues expressed CLL-derived immunoglobulins and an inducible BLNK adaptor in a BCR/BLNK-negative murine pre-B cell line and reported Ca^2+^ flux in the absence of an external BCR ligand. Interestingly, this signal was observed independently of BCR type (stereotyped vs non-stereotyped) and regardless of IGHV mutation status. Importantly, such Ca^2+^ flux was not observed after expression of immunoglobulins derived from other NHLs, suggesting a CLL-specific mechanism. Additional experiments revealed that the HCDR3 regions of CLL immunoglobulins recognized epitopes in the variable or constant regions of adjacent surface immunoglobulin molecules, providing an explanation for the inherent capacity of CLL BCRs to undergo autonomous interactions [69,70,71]. Consistent with cell-autonomous reactivity of CLL BCRs, a very recent study employing super-resolution microscopy provided further evidence of such interactions by visualizing oligomeric BCR nanoclusters on the surface of unstimulated CLL B cells [3]. 

Following the studies by Dühren-von Minden and colleagues, our group used the Eµ-TCL1-transgenic mouse model to evaluate the capacity of different types of transgenic BCRs to induce leukemia in vivo [72]. This study showed that B cells that express transgenic BCRs that become activated by low-affinity extrinsic autoantigens and/or cell-autonomous interactions enter into the leukemogenic process and become CLL cells, whereas B cells that express high-affinity transgenic BCRs do not undergo malignant transformation regardless of antigen form (soluble or membrane-tethered) or presentation (foreign or self). Subsequent studies using the Eµ-TCL1-transgenic mouse model and transgenic BCRs with other specificities reaffirmed these findings. In particular, the study of Hayakawa et al. showed that B cells expressing a transgenic low-affinity anti-Thy-1 BCR undergo malignant transformation only in the presence of the Thy-1 autoantigen, confirming that chronic stimulation with extrinsic low-affinity autoantigen can induce leukemia in vivo [73]. To further explore the role of high-affinity foreign antigens in driving leukemia development and progression, Jiménez de Oya and colleagues generated Eµ-TCL1 tg mice carrying gene-targeted immunoglobulin heavy chains from antibodies reactive with lymphocytic choriomeningitis virus or vesicular stomatitis virus [74]. Although these mice developed leukemias that express BCRs reactive with their cognate viral antigens, the presence or absence of the viral antigens had no influence on the rate of leukemia development or progression. Rather, the transgenic heavy chains preferentially paired with light chains that conferred to the leukemic BCRs the ability to cross-react with various external autoantigens or undergo cell-autonomous interactions. Taken together, the findings from these three studies provide in vivo evidence that self reactivity is a major driving force in CLL pathogenesis and suggest that only BCR signals of certain quality can promote the growth of the malignant cells.

The murine studies also suggested that the capacity of the leukemic cells to respond to external antigen can influence the aggressiveness of the disease and may account for the variability in the clinical course of CLL. In particular, we observed that leukemia developed more rapidly when the malignant cells expressed BCRs that generated a weak autonomous signal but responded strongly to stimulation with external antigen, compared to cells expressing BCRs that generated a strong autonomous BCR signal but did not respond or only weakly responded to external antigen stimulation [72]. Similar findings were presented by Minici and colleagues who investigated the strength of the autonomous interactions of human CLL BCRs belonging to two distinct stereotyped subsets [71]. Importantly, in the more indolent BCR subset #4, self-recognition was found to be tight and long-lived, while in the more aggressive BCR subset #2, self-association was found to be of low-affinity and shorter duration [71]. The explanation for this correlation is that strong self-recognition pushes the cells toward anergy, while a weaker self-recognition implies higher BCR availability to bind to external self-antigens and to respond to such stimulation.

The above findings also imply that the signals generated by cell-autonomous and cell-extrinsic interactions are not equivalent and may induce distinct cellular responses [75]. Cell-autonomous signals might provide a repetitive stimulation of low or intermediate strength, which may act as a continuous survival source for the leukemic cells. On the other hand, BCR stimulation with external ligand has been shown to increase the expression of the cell cycle regulators MYC, CCND2, and CDK4 and to increase the percentage of CLL cells in the G1 phase of the cell cycle, suggesting that interactions with external autoantigens may provide the initial stimulus required for leukemic cell proliferation [76,77,78]. Defining the downstream signaling pathways that are activated by cell-autonomous and cell-extrinsic interactions will be required to understand the exact nature of the cellular processes regulated by these two mechanisms of BCR pathway activation. Addressing this question is important in view of the possibility that different prognostic subgroups of CLL may be driven by different types of BCR signals and that these BCR signals may be differently targeted by available BCR signaling inhibitors. 

## 4. BCR Signaling in Diffuse Large B Cell Lymphoma

DLBCL is a heterogeneous neoplastic entity, characterized by a high mutational load: the implementation of next generation sequencing techniques revealed that the mean number of coding genome alterations exceeds 70 per single DLBCL case and identified over 150 genetic drivers [79,80]. Such genetic complexity is responsible for a very heterogeneous phenotype as well as for a significantly variable response to therapy. While the details of DLBCL pathogenesis are extensively reviewed elsewhere [81], we will mainly concentrate on the mechanisms of activation of the BCR pathway. 

Genomic, biochemical, and functional analyses performed in the last two decades have resulted in the identification of several different DLBCL subtypes. The first classification, made by the Staudt group [82], defined two distinct DLBCL subtypes, termed Germinal Center B cell-like (GCB) and Activated B cell-like (ABC) DLBCL, which represent lymphomas arising from different stages of lymphoid differentiation. As discussed below, the two subgroups differ profoundly when the mechanisms of BCR activation are concerned. Recently, these subgroups have been further subdivided based on combinations of recurrent genetic lesions harbored in the malignant cells, which presumably define different evolutionary trajectories leading to lymphomagenesis [83,84]. In parallel, another DLBCL classification has been proposed by the Shipp group, which distinguishes DLBCLs that depend on the BCR for their survival (BCR-dependent, containing both ABC and GCB DLBCLs) from DLBCLs that evolve through other pathogenetic mechanisms [85,86].

Pioneering evidence for the involvement of BCR signaling in ABC DLBCL came from the study of Davis et al., which revealed that signals departing from the BCR were crucial for the activation of the NF-κB pathway, described to be essential for the survival of ABC DLBCL cells [87]. Indeed, knockdown of BTK, CD79A, or IgM was selectively toxic for a substantial proportion of ABC DLBCL cell lines, pointing to the importance of BCR signaling for the constitutive activation of NF-κB. Furthermore, total internal reflection fluorescence (TIRF) microscopy revealed prominent BCR clusters on these ABC DLBCL cell lines that were not present in cell lines derived from GCB DLBCL, BL, or MCL. Importantly, these clusters were also present on BCR-stimulated but not on unstimulated normal B cells, suggesting that they reflect active proximal BCR signaling. Of note, a substantial proportion of these cases were shown to harbor heterozygous mutations in the CD79B ITAM domain that prevent recruitment of LYN and disrupt LYN-mediated endocytic internalization of the BCR. Importantly, this study also showed that CD79B mutations amplify the BCR signal but are unable to induce BCR clustering and activation on their own. The mechanism responsible for BCR activation in these cases was revealed in a subsequent study by the same group demonstrating that reactivity with self-antigens expressed on the same or adjacent cells was responsible for generating and maintaining this chronic active form of signaling [88]. 

Although these modalities of BCR activation are exquisitely reminiscent of the mechanisms of BCR activation in CLL cells, a noteworthy difference is the absence of CD79B mutations in the latter disease. This could suggest that the quality of the BCR signal is not the same in ABC DLBCL and CLL or that the two diseases employ different strategies in the context of similar biological phenomena. As recently suggested, the role of CD79 mutations in DLBCL may be to block the LYN-mediated induction of anergy [89], providing an additional explanation of why these mutations are frequently selected in ABC DLBCL. Of note, the IGHV4-34 gene, which is used by almost one third of all ABC DLBCL tumors, was found to associate in almost 40% of the cases with CD79B mutations [83,88]. This gene typically encodes for autoantibodies that react with cell surface glycoproteins and is expressed on a subset of peripheral blood B cell that express low levels of surface IgM and have other features of anergy [90]. Thus, by blocking BCR internalization and by enhancing BCR signaling, CD79B mutations may prevent induction of anergy, which in the case of CLL may be primarily overcome by co-stimulatory signals from the tumor microenvironment [91,92]. 

The mechanisms responsible for activation of the BCR pathway in GCB DLBCL until very recently remained unknown, despite the long-standing evidence that the BCR-proximal kinase SYK is activated in a substantial proportion of GCB DLBCL cell lines and primary GCB DLBCL tumor samples [93,94,95,96]. The BCR dependency of these GCB DLBCL lines was not detected in the initial RNA interference screens that revealed the BCR-dependence of the ABC DLBCL cell lines [87], presumably because of the incomplete knockdown of BCR subunits with this technique, but was confirmed more recently using a CRISPR/Cas9 genome editing approach [97]. Parallel biochemical studies revealed that only the PI3K/AKT pathway is activated downstream of the BCR in GCB DLBCL, in contrast to ABC DLBCL where both PI3K/AKT and NF-κB are activated [86]. Another important difference between the two subsets was the absence of BCR clusters and the much lower frequency of CD79B mutations in GCB as opposed to ABC DLBCL [87,98]. Moreover, replacement of the endogenous BCR with a BCR specific for a foreign antigen did not affect the growth of GCB DLBCL cells but inhibited the growth of ABC DLBCL cells, further suggesting that BCR signaling is antigen-independent in GCB DLBCL and antigen-dependent in ABC DLBCL [98]. 

To further examine the potential mechanisms for BCR activation in GCB DLBCL, we recently investigated the expression of several negative regulators of this pathway in a series of BCR-dependent and BCR-independent DLBCL cell lines [26]. Remarkably, all of the BCR-dependent GCB DLBCL cell lines (*n* = 6) and 2 of the 4 BCR-dependent ABC DLBCL cell lines showed absent or markedly reduced expression of the phosphatase SHP1. As pointed above, SHP-1 is the principal negative regulator of the BCR pathway and functions by dephosphorylating several BCR proximal signaling molecules, including CD79A, CD79B, SYK, BLNK, and CD19 [99,100]. Accordingly, these SHP-1 negative lines showed increased levels of phosphorylated SYK and BLNK which were reduced by SHP-1 re-expression, providing direct evidence that SHP1 deficiency is at least in part responsible for the constitutive activation of the BCR pathway in GCB DLBCL [26].

**Figure 3:**
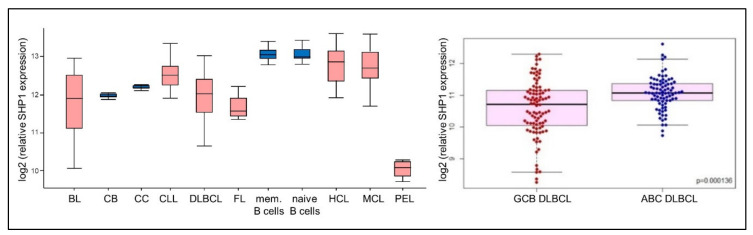
SHP1 expression in different B cell malignancies: (**left**) Comparison of SHP1 mRNA levels in normal and malignant B cell subsets analysed in dataset GSE2350 (CB, centroblasts; CC, centrocytes; mem. B cell, memory B-cells; HCL, Hairy Cell Leukemia; PEL, Primary Effusion Lymphoma). (**right**) Comparison of SHP1 expression levels in germinal center B-cell-like (GCB and Activated B cell-like (ABC) diffuse large B cell lymphoma (DLBCL) tumors analysed in dataset GSE4732.

Previous immunohistochemical studies had reported that SHP1 is downregulated in approximately 40% of primary DLBCL tumors [101,102]. These findings were further corroborated in our study, which showed substantially lower levels of SHP1 mRNA in primary DLBCL tumors compared to normal GC B cells in approximately half of the cases, with significantly more frequent downregulation in GCB compared to ABC DLBCL (Figure 3) [26]. Moreover, this analysis revealed that SHP1 is also frequently downregulated in a substantial proportion of BL, FL, and primary effusion lymphoma (PEL) tumors (Figure 3), suggesting that SHP1 deficiency could represent a common mechanism of BCR pathway activation in several B cell malignancies. 

The mechanisms responsible for SHP1 downregulation in DLBCL have still not been fully elucidated, but two recent studies reported the presence of inactivating SHP1 mutations in approximately 5% of primary DLBCL tumors [83,84]. Other causes of SHP-1 downregulation could include inactivating genetic lesions in the histone methyltransferase KMT2D and genetic lesions resulting in overexpression of the transcriptional repressor BCL-6, each of which is affected in approximately 30% of DLBCL tumors. KMT2D has been shown to positively regulate SHP-1 expression [103], while BCL6 represses its transcription [104], suggesting that part of the oncogenic activity of KMTD2D and BCL6 alterations could be related to SHP1 downregulation. 

**Figure 4:**
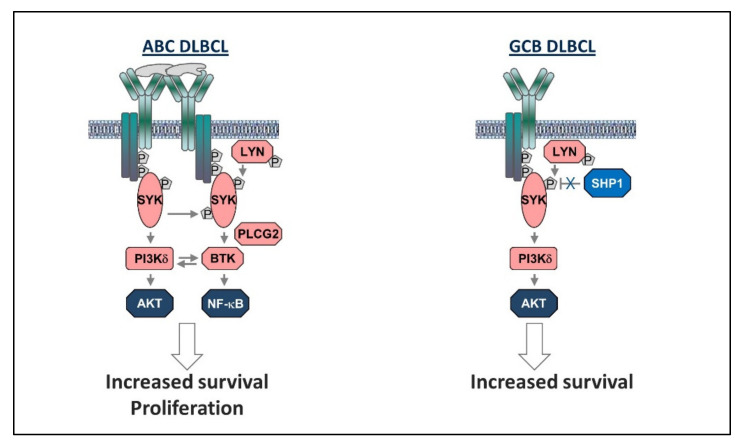
Mechanisms of B cell receptor (BCR) pathway activation in ABC and GCB DLBCL.

The findings described above suggest that two distinct mechanisms are responsible for activation of the BCR pathway in ABC and GCB DLBCL, respectively: an antigen-dependent mechanism resulting in a “chronic active” BCR signal that activates both NF-κB and PI3K and an antigen-independent mechanism resulting in an exaggerated “tonic” BCR signal that activates only PI3K. The different quality of the signals generated by these two mechanisms could potentially be explained by quantitative differences in the levels of SYK activation. In an earlier study, we showed that in unstimulated GCB DLBCL cells, SYK is primarily phosphorylated on the regulatory tyrosine (Y) at position 352, whereas phosphorylation of the regulatory tyrosines at 525/526 becomes mainly detectable following BCR crosslinking with an external ligand [95]. Phosphorylation of Y352 and YY525/526 represent two consecutive stages in SYK activation: phosphorylation of Y352 by SRC family kinases is the first stage, allowing SYK to adopt an open, catalytically active configuration, whereas trans-autophosphorylation of YY525/526 occurs in the second stage, resulting in stabilization of the activation loop and increased kinase activity (Figure 4). Importantly, experiments with phosphomimetics corresponding to these two stages of SYK activation revealed that phosphorylation of YY525/526 is associated with a drastic increase in PLCγ2 activation and a more modest increase in AKT activation. Considering that PLCγ2 is required for activation of NF-κB, this could provide an explanation for the higher NF-κB activity in BCR-dependent ABC compared to BCR-dependent GCB DLBCL cell lines. It is also worth noting that experiments with the IL-3 dependent murine B cell line BaF3 showed that only the fully activated SYK phosphomimetic induces growth-factor independent cell proliferation, whereas both phosphomimetics increased cell survival [95]. These findings suggest that the consequences of BCR pathway activation in ABC and GCB DLBCL are different and may provide a potential explanation for the different response rates to ibrutinib treatment [105]. 

## 5. BCR Signaling in Burkitt Lymphoma

BL in an aggressive germinal center B-cell malignancy divided into three subtypes: endemic, prevalent in young African children and associated with EBV infection; immunodeficiency related, mainly associated with HIV infection; and sporadic. Gene expression profiling studies have revealed that BL cells have a distinct molecular signature consistent with derivation from dark zone GC B cells [106].

A hallmark feature of BL are translocations of the MYC oncogene resulting in aberrant MYC activation. In over 80% of cases, the translocation partner is the immunoglobulin heavy chain locus on chromosome 14, whereas in the remaining cases, MYC is translocated in the κ or λ light chain locus on chromosome 2 and 22, respectively. Importantly, the MYC translocation always spares the productive IGHV and IGLV genes that are used to construct the BCR, indicating that BL cells depend on signals from the BCR for growth or survival [107]. 

Initial evidence for BCR involvement in BL lymphomagenesis came from the study of Schmitz and colleagues, who reported recurrent mutations in the transcription factor TCF3/E2A and its negative regulator ID3 in 70% of sporadic BL tumors [108]. These mutations relieve TCF3 from the negative influence of ID3 and promote its constitutive activity, resulting in changes in expression of numerous transcriptional targets of TCF3. Importantly, these changes included upregulation of IGHV and IGLV gene expression and repression of SHP1, suggesting a similar mechanism of BCR pathway activation to the one previously described in GCB-DLBCL. Consistent with this possibility, inhibition of PI3K/AKT signaling and apoptosis induction was observed in the majority of BL cell lines following knockdown of TCF3, CD79A, or SYK. 

Further evidence for a role of the BCR/PI3K/AKT pathway in BL pathogenesis was provided by Varano and colleagues, who demonstrated in a MYC transgenic mouse model of BL that BCR-negative lymphoma cells can proliferate and survive in vitro but exhibit a competitive growth disadvantage compared to their BCR-positive counterparts [109]. The competitive growth advantage of the BCR-positive BL cells was driven mainly by PI3K/AKT-dependent inhibition of the kinase GSK3β, which facilitated G1 to S phase cell cycle transition and increased cell survival. These effects were more pronounced under conditions of nutrient deprivation, suggesting that BCR signals increase the metabolic fitness of the malignant cells. 

Similar findings were reported by another group using the human BL cell line Ramos, which showed that the signals that provide the competitive advantage to BCR-positive BL cells involve interactions between LYN, SYK, CD19, and CD79B [110]. Interestingly, the survival of BCR-dependent GCB-DLBCL cell lines also requires LYN and CD19 [97], providing another similarity between BL and GCB-DLBCL and pointing to a common mechanism of PI3K/AKT activation downstream of the BCR in these two diseases. It should be noted, however, that another recent study reported that BL cells do not have a hyperactivated PI3K-AKT pathway and are not sensitive to AKT knockdown or inhibition in contrast to GCB DLBCL cells [111]. These data argue against a growth-promoting role for the PI3K/AKT pathway in BL and underline the need for additional studies on the role of the BCR pathway in the pathogenesis of this disease. 

## 6. BCR Signaling in Follicular Lymphoma

FL is an indolent B cell malignancy derived from GC B cells [112]. Its hallmark feature is the t(14;18) (q32;q21) translocation, which is present in approximately 90% of the patients. The translocation brings the BCL-2 gene into the IGHC gene locus, resulting in constitutive BCL2 expression and apoptosis evasion. As in the case of BL, the translocation always spares the productively rearranged IGHV allele, allowing for the expression of a functional BCR. 

An important characteristic of FL is the ongoing SHM, with the consequent acquisition of novel mutations and intraclonal variation of the IGHV and IGLV genes. The random introduction of these mutations would be expected to result in stop codons and loss of immunoglobulin expression. However, FL tumors always maintain surface immunoglobulin, indicating a selective force that favors retention of a functional BCR. Accordingly, treatment of FL patients with anti-idiotype antibodies was associated with the outgrowth of escape variants that still expressed a functional BCR but were no longer recognized by the anti-idiotype antibody because of SHM in the targeted V region sequence [113]. Moreover, in vitro studies have shown that pharmacological inhibition or RNAi-mediated knockdown of SYK in FL cell lines results in inhibition of the PI3K/AKT/mTOR pathway, with consequent growth arrest and reduced invasive capacity, further implicating the BCR pathway in the pathogenesis of FL [114,115]. 

Available evidence suggests that three mechanisms may be responsible for the activation of the BCR pathway in FL. Studies from the Stevenson group have revealed that 80% of FL patients carry SHM-introduced N-glycosylation sites in the functional IGHV gene, most of which are found in the complementarity-determining regions [116]. More recent next-generation IGHV sequencing studies confirmed these data, observing acquisition of N-glycosylation sites in 96% of FL subclones, with loss of negative subclones in successive events of disease progression [117]. The N-glycosylation sites represent cues for the addition of “high-mannose” glycans, which bind to the lectin DC-SIGN that is overexpressed on M2 macrophages and dendritic cells in FL lymph nodes [118]. This interaction has been shown to induce a continuous low-level BCR signal in the tumor cells, resulting in SYK, ERK, and AKT activation, Ca^2+^ flux, and induction of MYC expression [118,119,120]. 

Another potential mechanism of BCR pathway activation in FL is binding to self-antigens. Using permeabilized human HEp-2 cells as a screen for human tissue antigens, Sachen and colleagues tested 98 FL immunoglobulins and observed autoreactivity in 26% of the cases [121]. For one tumor immunoglobulin, the self-antigen was identified as myoferlin, a protein associated with cell and nuclear membranes. Interestingly, reactivity with myoferlin was also observed in another study that used a combinatorial peptide library to identify ligands for three FL immunoglobulins [122]. The myoferlin peptide sequence that was recognized by one of these antibodies was found to be identical to a sequence in a surface protein from *Streptococcus mitis* and *Pneumocystis jirovecii*, suggesting crossreactivity with foreign antigens.

Consistent with these findings, another study reported reactivity of FL-derived immunoglobulins with HEp-2 cells in 11% of the 217 investigated cases [123]. A considerable proportion of these cases reacted with vimentin, which is an intracellular filament protein that is expressed on the surface of activated and apoptotic T cells, macrophages, neutrophils, and platelets. Interestingly, the percentage of cases reacting with the N-terminal region of vimentin was even higher than the percentage of cases reacting with HEp-2 cells, indicating the existence of different conformational epitopes that can be recognized by the tumor immunoglobulins. 

Apart from aberrant BCR engagement, the addiction of FL to BCR-related pathways could potentially be caused by mutations in molecules that transduce the BCR signal. Indeed, whole exome sequencing (WES) analysis identified recurrent mutations in the interconnected BCR and CXCR4 pathways in almost 45% of FL patients, including mutations in BTK, SYK, BLNK, CD22, PLCG2, EGR1, and EGR2 [124]. Although the functional consequences of these mutations are still unclear, an effect on BCR signal transduction would be expected. Perhaps equally relevant is the fact that KTM2D is mutated in approximately 60% of the FL tumors [112,124] and may account for the frequent downregulation of SHP1 that was previously mentioned. Accordingly, SHP-1 deficiency could cooperate with expression of mannosylated immunoglobulins to amplify the BCR signal generated by mannose-binding lectins, as already proposed [118]. Considering that N-glycosylation sites are also present in approximately 40% of DLBCL immunoglobulins [116], it is possible that these two mechanisms cooperate in activating the BCR pathway also in GCB DLBCL. 

## 7. BCR Signaling in Mantle Cell Lymphoma

A hallmark of MCL is the t(11;14)(q13;q32) translocation which brings the CCDN1 gene into the IGHC gene locus, resulting in cyclin D1 overexpression. Other frequent genetic alterations include mutation or deletion of ATM and TP53 and copy number alterations that typically affect genes related to cell cycle regulation, DNA damage response, and cell survival, including CDKN2A, RB1, MYC, CDK4, or BCL2. In addition, recurrent mutations in chromatin modifiers and genes belonging to the NOTCH and NF-κB pathways have been identified in a substantial proportion of cases (reviewed in Reference [125]).

The malignant cells typically express unmutated IGHV genes (>98% homology to germline sequence), although around 30% of cases show a higher level of SHM [126,127]. In favor of an antigen-driven lymphomagenesis, expression of stereotyped BCRs has been reported in approximately 10% of MCL cases [128,129]. Additional evidence for a potential role of the BCR pathway in the pathogenesis of MCL was provided by phospho-proteomic analysis of MCL cell lines and primary tumor samples demonstrating constitutive activation of multiple BCR signaling molecules, including CD79B, LYN, SYK, BLNK, BTK, and PLCγ2 [130]. Treatment of these cell lines with SYK inhibitors induced cell cycle arrest and apoptosis, suggesting that BCR signals regulate the proliferation and survival of the malignant cells [131]. A growth inhibitory effect was also observed with ibrutinib against a subset of MCL cell lines characterized by activation of the cannonical NF-κB pathway [132]. In contrast, ibrutinib was ineffective against MCL cells lines with constitutive activation of the noncannonical NF-κB pathway caused by genetic lesions in the regulatory components TRAF2 and BIRC3.

A study from Wiestner’s group also revealed the existence of two MCL subsets that differ with respect to the mechanism of NF-κB activation. In this study, transcriptome analysis of lymph node and peripheral blood MCL cells identified a subset with higher expression of BCR and NF-κB target genes in lymph node MCL cells and a subset with equal expression of BCR and NF-κB target genes in the two cell populations [133]. Cases from the first subset showed higher expression of phosphorylated SYK, PLCγ2, AKT, ERK, and the NF-κB p65 subunit in lymph node compared to peripheral blood MCL cells, consistent with a BCR-dependent mechanism of NF-κB activation.

The mechanisms that activate the BCR pathway in MCL have still not been fully elucidated, although most data indicate a role for autoantigen stimulation. Similar to FL, the tumor immunoglobulins in MCL have been reported to bind HEp-2 antigens, including vimentin, in about one-third of the cases [123]. In another study, reactivity with the autoantigen low-density lipoprotein receptor-related protein-associated protein 1 (LRPAP1) was identified in 36% (10/28) of investigated tumor immunoglobulins [134]. In addition, given the similarities with CLL and the finding that MCL cells frequently present neoantigenic peptides derived from the lymphoma immunoglobulin heavy- or light-chain variable regions to idiotype-specific T cells [135], it remains possible that the BCR pathway is activated in a subset of cases by cell-autonomous BCR interactions. Such interactions were not detected by Dühren-von Minden et al. in their seminal study [69], but the small number of investigated MCL immunoglobulins in that study does not entirely exclude this possibility.

## 8. BCR Signaling in Marginal Zone Lymphoma

MZL is a B cell malignancy comprised of three distinct entities: the extranodal MZL of mucosa-associated lymphoid tissue (MALT lymphoma), splenic MZL (SMZL), and nodal MZL (NMZL). The cells of origin are marginal zone B cells, which act as a first line of defense against infectious agents and are responsible for the mounting of a rapid, innate-like antibody response against both T cell–dependent and T cell–independent antigens. The three MZL subtypes share common genetic lesions and deregulated pathways and present subtype-specific alterations. The NF-κB pathway is activated in approximately 50% of cases from all three subsets by loss-of-function lesions in the negative regulators TNFAIP3 (A20), BIRC3, and TRAF3, or translocations resulting in deregulated expression of the positive regulators MALT1 or BCL10. Other frequent genetic lesions include mutations that activate the NOTCH pathway, which are present in approximately 40% of SMZL and NZML cases but in less than 5% of MALT lymphomas, and mutations that inactivate the transcription factor KLF2, which are detected in 20–40% of SMZL and 20% of NZML cases (reviewed in Reference [136]). 

MZL was one of the first B cell malignancies with evidence for a role of chronic antigen stimulation in the pathogenesis of the disease. Specific disease entities are frequently associated with certain chronic bacterial or viral infections, such as gastric MALT lymphomas with *Helicobacter pylori*, SMZL with *Hepatitis C virus* (HCV), ocular adnexal MALT lymphomas with *Chlamydia psittaci*, cutaneous MALT lymphomas with *Borrelia burgdorferi*, and immunoproliferative small intestine disease with *Campylobacter jejuni* infection [137,138,139,140,141]. The pathogenic role of chronic antigen stimulation in these MZL entities is further supported by the capacity of antimicrobial drugs to induce tumor regression upon eradication of the infectious agent in patients at early stages of the disease [142,143,144]. Beside infection, chronic antigen stimulation in the context of certain autoimmune disorders may also contribute to the development of MZL, given the association of Sjögren syndrome and Hashimoto’s thyroiditis with MALT lymphomas of the salivary gland and thyroid, respectively [145,146].

The malignant B cells in MZL typically express somatically mutated IGHV genes with a pattern of mutations consistent with antigen selection. In addition, biased IGHV gene usage and expression of stereotyped BCRs is frequently observed, further hinting to a BCR-driven process. The most frequently overrepresented genes include IGHV1-69, which is predominantly expressed in HCV-associated MZLs and salivary gland and gastric MALT lymphomas, and IGHV1-02, which is expressed in more than one third of splenic MZLs not associated with HCV infection. In addition, the IGHV3-30, IGHV3-23, IGHV3-7, and IGHV4-34 genes are frequently overrepresented, particularly in splenic MZL and gastric and orbital adnexal MALT lymphomas [137,147,148,149,150,151,152,153].

The IGHV1-69 immunoglobulins in MZL often contain the IGKV3-20 light chain and typically display rheumatoid factor (RF) activity [137,154]. RF activity has also been reported for MZL immunoglobulins encoded by other overrepresented IGHV genes, including the stereotypic combination IGHV3-7/IGKV3-15 [137,155]. Moreover, reversion of IGHV and IGKV mutations in these RFs to germline configuration reduced the affinity for IgG, suggesting that the tumor cells were selected for their capacity to bind to IgG or IgG-containing immune complexes [155]. In addition to RF activity, reactivity of MZL immunoglobulins with other self antigens has been reported, including insulin, thyroglobulin, galactosidase, galectin-3, stomach extract, and other human tissue antigens [151,156,157]. This polyreactive binding pattern resembles the reactivity of CLL immunoglobulins, although the affinity of the latter is considerably lower. Interestingly, despite the frequent association of MZL with chronic infections, most evidence suggests that the tumor imunoglobulins do not bind directly to the microbial antigens [137,149]. These findings suggest that the BCR pathway in MZL is primarily activated by autoantigens and indicate that the role of the infectious agents may be to provide costimulatory signals rather than activate the BCR pathway [158]. 

The signaling pathways that are activated downstream of the BCR in MZL cells have not been investigated in detail, but analysis of primary tumor samples by phospho-flow cytometry demonstrated constitutive activation of SRC family kinases, SYK, PLCγ2, and p65 NF-κB [159]. Interestingly, some of the more frequent genetic defects in MZL can potentially augment canonical NF-κB activation induced by stimulation of the BCR. These most notably include inactivating mutations in the negative regulator TNFAIP3 (A20), which are detected in ~30% of MALT lymphomas and in 10–15% of splenic and nodal MZLs, and inactivating mutations in the transcription factor KLF2, which have been reported in 20–40% of SMZL and in 17% of NZML (reviewed in References [160,161]. In contrast, other frequent genetic defects, such as the t(1;14)(p22;q32), t(14;18)(q32;q21), and t(11;18)(q21;q21) translocations, which are detected in over 25% of gastric and over 50% of lung MALT lymphomas, result in overexpression of BCL10 or MALT1 and constitutive activation of the canonical NF-κB pathway [161]. Interestingly, these translocations are not seen in cases with RF BCRs and have been associated with resistance to *H. pylori* eradication therapy, suggesting that they may define a subset of MALT lymphomas that do not depend on BCR-mediated NF-κB activation for their expansion [137]. 

## 9. BCR Signaling in Waldenstrom Macroglobulinemia

WM is defined as lymphoplasmacytic lymphoma associated with monoclonal IgM. It is a rare and indolent B-cell malignancy, characterized by the infiltration of cells in the bone marrow and extramedullary sites. Studies analyzing the genomic landscape of WM have identified mutations in the Toll-like receptor (TLR)-adaptor protein MYD88 and the chemokine receptor CXCR4 (present in >90% and 30–40% of cases, respectively) as the principal hallmarks of the disease. In addition, deletions in chromosome 6q21-25 occur in about 50% of patients and affect the expression of several genes involved in BCR signaling, including the BTK inhibitor IBTK, the transcription factor FOXO3, and the NF-κB regulators TNFAIP3 and HIVEP2. Other recurrent genetic defects in WM include mutations in CD79A and CD79B, which have been reported in 7–15% of the cases (reviewed in Reference [162]). 

Some of the initial evidence for a pathogenic role of the BCR in WM came from its association with several autoantibody-mediated autoimmune disorders, such as cold agglutinin disease, type II mixed cryoglobulinemia, and certain polyneuropathies (reviewed in Reference [163]). The finding that type II mixed cryoglobulinemia can evolve into WM and that the tumor immunoglobulins in these cases typically bind to HCV-containing immune complexes further substantiated this possibility [164,165]. The tumor immunoglobulins in HCV-associated WM were shown to express a biased IGHV gene repertoire with predominant usage of the RF-encoding IGHV1-69/IGKV3-20 combination, consistent with antigen-driven selection [166]. Subsequent studies investigating WM without HCV infection also showed a restricted IGHV repertoire, although with overrepresentation of other genes, such as IGHV3-23, IGHV3-7, and IGHV3-74 [167,168,169]. 

Additional support for an involvement of the BCR pathway in the pathogenesis of WM came from observations that a number of BCR signaling molecules, including SFK, SYK, BTK, BLNK, PLCγ2, ERK, AKT, and NF-κΒ, are constitutively activated in primary WM tumor cells [170]. Moreover, treatment of WM cell lines with BTK or SYK inhibitors resulted in cell cycle arrest, apoptosis, and inhibition of AKT and ERK signaling [171,172]. Interestingly, both BTK and SYK have been shown to directly interact with mutated MYD88 in WM cells [171,173]. Moreover, knockdown of MYD88 or use of a MYD88 inhibitor reduced the levels of activated SYK, BTK, AKT, and STAT3 in these cells, suggesting a novel mechanism of BCR pathway activation that involves direct cooperation with the TLR pathway [173]. A similar mechanism has been described in ABC DLBCL, where a signaling complex composed of MYD88, TLR9, and IgM has been identified in the endolysosomal compartment of MYD88-mutated cells [97]. This complex has been shown to drive the sustained NF-κB activity in ABC DLBCL cells and presumably mediates the same effect in WM cells.

## 10. BCR Inhibitors

The identification of the BCR as a major therapeutic target in various B cell malignancies resulted in the clinical testing and approval of multiple drugs that block signaling through this receptor. Most of these drugs target BTK or PI3K, although inhibitors of SYK, SRC family kinases, and mTOR have also demonstrated activity in clinical trials (Table 1). The primary mechanism of action of these drugs is inhibition of BCR-induced proliferation and survival signals [174,175,176,177], although inhibition of other BCR-regulated processes has also been shown to contribute to the activity of these drugs, particularly in CLL. Specifically, inhibition of BCR-mediated integrin activation has been shown to reduce the adhesion of the leukemic cells to extracellular matrix and cell surface adhesion proteins, whereas inhibition of BCR-mediated chemokine secretion has been shown to result in reduced recruitment of T cells and monocytes [178,179,180,181,182]. These effects reduce the retention of the leukemic cells in the nurturing lymph node microenvironment and prevent them from receiving growth and survival signals from accessory cells. In addition, part of the activity of these drugs has been attributed to their capacity to interfere with signaling through other receptors that regulate leukemic cell migration, proliferation, and survival, including CD40L, BAFF, IL-4, CXCR4, and TLR9 [29,183,184]. These effects would be expected to further deprive CLL cells of growth and survival signals in the microenvironment, resulting in “death by neglect” [185]. The importance of these non-BCR effects on clinical responses to BCR inhibitor treatment in B cell malignancies other than CLL are less certain at present, although similar effects on malignant cell migration and adhesion have been reported in MCL and WM [30,186].

### 10.1. SYK Inhibitors

Fostamatinib was the first BCR inhibitor that was tested in B cell malignancies, demonstrating potent preclinical activity in DLBCL and CLL [49,94,174]. A subsequent phase 1/2 clinical trial in patients with relapsed or refractory (R/R) B cell malignancies showed an objective response rate (ORR) of 55% for CLL, of 22% for DLBCL, of 10% for FL, and of 11% for MCL [196]. Another phase 2 clinical trial conducted only in patients with R/R DLBCL showed an ORR of 3%, indicating low single-agent activity of fostamatinib in DLBCL [217]. Similar results were obtained with the more selective SYK inhibitor entospletinib, which showed a 61% ORR in patients with R/R CLL and a more modest effect in other B cell malignancies, including a 35.3% ORR in LPL/WM, 17.9% in MCL, 17.1% in FL, 11.8% in MZL, and no responses in DLBCL [197,204,208]. Entospletinib also demonstrated clinical activity in R/R CLL patients that had received prior treatment with a BTK or PI3Kδ inhibitor, with 32.7% of patients responding to treatment, including 2 of 8 patients with Richter Transformation [198]. Cerdulatinib, another SYK inhibitor that is currently under development, also showed promising results for CLL and FL but not in DLBCL or MCL in a phase 1 clinical trial on patients with B cell malignancies [199]. Altogether, these data suggest that SYK inhibitors are active mainly in CLL, with more modest activity in other B cell malignancies.

### 10.2. BTK Inhibitors

Ibrutinib is a small molecule inhibitor that irreversibly inhibits BTK by covalently binding to cysteine 481 in the ATP-binding pocket. In vitro studies showed that ibrutinib mainly inhibits the BTK/PLCG2/PKC/NF-κB axis [218], although inhibitory effects on AKT and ERK have also been reported [27]. Ibrutinib was initially tested in a phase 1 study of patients with R/R B cell malignancies, which showed an ORR of 69% in CLL, of 78% in MCL, of 75% in WM, of 38% in FL, of 29% in DLBCL, and of 25% in MZL [189]. These remarkable results encouraged a phase 1b/2 study in 85 heavily pretreated R/R CLL patients, displaying an ORR of 71%. The ORR further increased after a 3-year follow-up, rising to 89% [219]. Ibrutinib also displayed a high ORR in phase 2 studies of MCL (68%), WM (91%), and MZ (48%%), resulting in its subsequent approval in these disease settings [209,215,220]. Lower activity was observed in phase 2 clinical trials of FL (ORR 38%) and DLBCL (ORR 25%), with the majority of the responding patients belonging to the ABC DLBCL subset (ORR of 37% in ABC DLBCL and 5% in GCB DLBCL) [105,205]. Notably, ibrutinib inhibits not only BTK but also other kinases such as ITK, TEC, EGFR, JAK3, ErbB, and SRC family kinases. Inhibition of some of these kinases has been shown to cause some of the side effects of ibrutinib, prompting the development of more selective BTK inhibitors. Acalabrutinib, a more specific molecule, in phase 2 studies of R/R CLL and R/R MCL displayed an ORR of 95% [190] and 81% [221], respectively, leading to its approval for treatment of patients with MCL that had received at least one prior line of therapy. Tirabrutinib, another more selective BTK inhibitor, also displayed considerable activity in patients with CLL and MCL, with 96% and 92% of patients responding to treatment, respectively. Tirabrutinib also displayed similar activity as ibrutinib in DLBCL, with an ORR of 35% in patients with non-GCB DLBCL [191]. 

### 10.3. PI3K Inhibitors

Idelalisib is an orally available reversible inhibitor of PI3K with 30–400 times greater selectivity for PI3Kδ compared to PI3Kα, PI3Kβ, and PI3Kγ. Phase 1 trials in R/R CLL and R/R MCL patients showed ORRs of 72% and 40%, respectively [192,210], while a phase 2 trial in patients with relapsed indolent B cell malignancies resulted in ORRs of 61% in CLL, of 54% in FL, of 47% in MZL, and of 80% in LPL/WM [193]. A subsequent phase 3 study of idelalisib in combination with rituximab in relapsed CLL displayed an ORR of 81% [222]. These trials led to the approval of idelalisib as a single agent in FL and SLL patients with at least 2 prior therapies and in combination with rituximab for second line treatment of CLL. Several second-generation PI3K inhibitors have more recently been developed, including the PI3Kγ/δ inhibitor Duvelisib and the PI3Kα/δ inhibitor Copanlisib. In a phase 2 trial of patients with refractory indolent NHL, duvelisib treatment resulted in ORRs of 67.9% in SLL, of 42.2% in FL, and of 38.9% in MZL [207], whereas an ORR of 74% was reported in a phase 3 trial of R/R CLL [194]. Copanlisib has also shown promising results in phase 2 trials of patients with R/R lymphoma, resulting in ORRs of 75% in SLL, of 59% in FL, of 70% in MZL, and of 64% in MCL [195,211]. More recently, copanlisib was tested in a phase 2 trial of patients with relapsed/refractory DLBCL, demonstrating ORRs of 31.6% and 13.3% in ABC and GCB DLBCL patients, respectively [19]. 

## 11. Therapeutic Implications 

The clinical data accumulated over the last decade demonstrate remarkable therapeutic efficacy of BCR inhibitors in patients with various B cell malignancies. Most of these data were obtained with the BTK inhibitor ibrutinib and the PI3Kδ inhibitor idelalisib, which induce clinical responses in the vast majority of patients with CLL, MCL, and WM and in approximately half of the patients with FL and MZL (Table 1). In addition, approximately one third of patients with ABC DLBCL initially respond to treatment with ibrutinib or the PI3Kα/δ inhibitor copanlisib, whereas responses are infrequent in patients with GCB DLBCL [19,105]. No clinical studies have been reported yet with BCR inhibitors in BL except for one case report [223]. Overall, the response rates in the various B cell malignancies largely correlate with the proportion of cases that have an activated NF-κB pathway downstream of the BCR (Table 2). As described previously, the majority of CLL, MCL, and WM cases demonstrate high NF-κB activity that can be reduced by treatment with a BCR inhibitor [133,171,176]. The proportion of such cases is lower in MZL and ABC DLBCL, which is consistent with the lower response rates in these diseases. Constitutive activation of the p65 subunit of NF-κB has also been detected in approximately 20% of primary FL tumors, although this has not yet been correlated with sensitivity to BCR inhibitor treatment [224]. 

In the majority of cases that do not respond to BCR inhibitor treatment, the NF-κB pathway is not activated or is activated by BCR-independent events. The latter mechanism appears to be particularly frequent in MZL and ABC DLBCL, where the NF-κB pathway is activated either by BTK-distal events, such as genetic lesions in BCL10, MALT1, and CARD11, or by genetic lesions in the non-canonical pathway, such as mutations in BIRC3 and TRAF3. Such mutations can be detected, although less frequently, also in MCL and CLL and have been associated with resistance to BTK-inhibitor treatment [105,132]. 

As previously discussed, the majority of FL, GCB DLBCL, and BL tumors have low NF-κB activity despite evidence for constitutive BCR activation. A major difference with respect to tumors with high NF-κB activity is the mechanism of BCR pathway activation. In FL, GCB DLBCL, and BL, available data suggest that the BCR pathway is activated by antigen-independent mechanisms that involve interactions with mannose-binding lectins and/or deficiency of the negative regulator SHP1. These mechanisms have been shown to induce signals of low intensity that are sufficient to activate ERK, AKT, and NFAT but apparently do not reach the threshold required for NF-κB activation [26,108,118,119,120,225]. Activation of NF-κB requires a large transient increase in intracellular Ca2+, such as occuring following acute engagement of the BCR with an external antigen [225]. Binding of the tumor immunoglobulins to autoantigens could provide such a signal, particularly in U-CLL, MZL, and WM, where the malignant cells frequently express autoreactive BCRs. The mechanism responsible for the activation of the NF-κB pathway in M-CLL is still unclear, considering that cell autonomous BCR–BCR interactions would not be expected to induce the high-amplitude Ca2+ elevations required for NF-κB activation. In this subset, it remains possible that the therapeutic activity of the kinase inhibitors is primarily mediated by BCR-independent mechanisms, such as inhibition of leukemic cell migration and adhesion. 

Clinical trials in tumors with an activated BCR pathway and low NF-κB activity, such as GCB DLBCL, have shown very modest efficacy of BCR inhibitors as single agents. However, such tumors typically display increased basal activity of SYK, PI3K, AKT, and GSK3, which regulate the expression of several BCL-2 family proteins, including MCL-1, BIM, and HRK [26,86,226,227]. Notably, treatment of such tumors with SYK, PI3K, and to a lesser extent BTK inhibitors results in reduced expression of the antiapoptotic protein MCL-1 and increased expression of the proapototic proteins BIM and HRK [26,227]. These changes induce relatively little apoptosis on their own but can sensitize the malignant cells to cytotoxic drugs, such as venetoclax. Together, these data suggest that combinations of BCR inhibitors with venetoclax deserve further study in tumors characterized by an activated BCR and overexpression of BCL2. 

Although the different BCR inhibitors have still not been compared in randomized clinical trials, available data indicate higher response rates and more durable responses with BTK inhibitors compared to PI3Kδ and particularly compared to SYK inhibitors (Table 1). This is somewhat surprising considering that SYK and PI3Kδ are positioned more proximally than BTK, and therefore, their inhibition would be expected to result in more complete inhibition of the BCR pathway, at least with respect to PI3K/AKT signaling. One possible explanation for the greater activity of BTK inhibitors is the more persistent inhibition of the BCR signal caused by the covalent interaction of these drugs with cysteine 481 in the ATP binding domain of the enzyme. In support of this possibility, mutations in cysteine 481 that alter the irreversible covalent binding of ibrutinib to a reversible interaction lead to drug resistance [228,229]. Moreover, a recent study comparing twice a day and once a day dosing of acalabrutinib in patients with CLL showed that the lower BTK occupancy in the latter cases was associated with a lower degree of BCR pathway inhibition and a lower ORR [230]. Collectively, these data suggest that the durability of BCR pathway inhibition is an important determinant of clinical response to drugs that target the BCR pathway.

## 12. Conclusions

The substantial differences in response rates and response duration to BCR inhibitor treatment suggest variable dependencies on BCR signaling and distinct mechanisms of BCR pathway activation between different B cell malignancies. In tumors with highest response rates, the BCR is typically activated by antigen-dependent mechanisms, resulting in the activation of multiple downstream signaling pathways, including NF-κB and PI3K/AKT. In tumors where the BCR pathway is activated by antigen-independent mechanisms, the NF-κB pathway is not activated or is activated by other mechanisms and responses to single-agent BCR inhibitor treatment are considerably lower. However, targeting the BCR pathway in such tumors leads to inhibition of PI3K/AKT signaling and changes in the expression of apoptosis regulatory proteins that can sensitize the malignant cells to cytotoxic drugs. Combinations of BCR inhibitors with cytotoxic agents such as venetoclax may provide a therapeutic benefit also in these patients and warrant future clinical trials. Future work should focus on the development of clinically useful biomarkers to identify patients with an activated BCR pathway and to select the optimal BCR inhibitor for individual patient treatment. 

## Figures and Tables

**Table 1:** Responses to BCR inhibitor treatment in different B cell malignancies.

Patient Population	Target	Drug	ORR	CR Rate	PFS	Ref.
CLL/SLL (R/R *)	BTK	Ibrutinib	71%	2%	88% at 6 months	[187]
CLL/SLL (R/R)	BTK	Ibrutinib	94%	9%	76% at 30 months	[188]
CLL/SLL (R/R)	BTK	Ibrutinib	69%	13%	n/a	[189]
CLL (R/R)	BTK	Acalabrutinib	95%	0	90% at 16 months	[190]
CLL (R/R)	BTK	Tirabrutinib	96%	n/a	median 29 months	[191]
CLL (R/R)	PI3Kδ	Idelalisib	72%	0	median 15.8 months	[192]
SLL (R/R)	PI3Kδ	Idelalisib	61%	n/a	n/a	[193]
CLL/SLL (R/R)	PI3Kα/δ	Duvelisib	74%	0.6%	median 13.3 months	[194]
SLL (R/R)	PI3Kα/δ	Copanlisib	75%	0	n/a	[195]
CLL/SLL (R/R)	SYK	Fostamatinib	55%	0	median 6.4 months	[196]
CLL (R/R)	SYK	Entospletinib	61%	0	median 13.8 months	[197]
CLL (R/R)	SYK	Entospletinib	33%	0	median 5.6 months	[198]
CLL/SLL (R/R)	SYK/JAK	Cerdulatinib	38%	0	n/a	[199]
CLL/SLL (R/R)	mTOR	Everolimus	18%	0	median 5.1 months	[200]
CLL/SLL (R/R)	mTOR	Everolimus	19%	0	n/a	[201]
CLL/SLL (R/R)	SFKs	Dasatinib	60%	13%	median 7.5 months	[202]
CLL (TN*)	BTK	Ibrutinib	85%	26%	90% at 18 months	[188]
CLL (TN*)	BTK	Ibrutinib	86%	4%	not reached	[203]
ABC-DLBCL (R/R)	BTK	Ibrutinib	37%	10%	median 2 months	[105]
GCB-DLBCL (R/R)	BTK	Ibrutinib	5%	0	median 1.3 months	[105]
DLBCL (R/R)	BTK	Tirabrutinib	35%	9%	median 1.7 months	[191]
DLBCL (R/R)	PI3Kδ	Idelalisib	0%	0%	0	[18]
ABC-DLBCL (R/R)	PI3Kα/δ	Copanlisib	32%	21%	median 4.3 months	[19]
GCB-DLBCL (R/R)	PI3Kα/δ	Copanlisib	13%	3%	median 6.0 months	[19]
DLBCL (R/R)	SYK	Fostamatinib	22%	6%	median 2.7 months	[196]
DLBCL (R/R)	SYK	Entospletinib	0	0	median 1.5 months	[204]
DLBCL (R/R)	SYK/JAK	Cerdulatinib	6%	0	n/a	[199]
FL (R/R)	BTK	Ibrutinib	38%	19%	n/a	[189]
FL (R/R)	BTK	Ibrutinib	38%	13%	median 14 months	[205]
FL (R/R)	PI3Kδ	Idelalisib	54%	n/a	n/a	[193]
FL (R/R)	PI3Kδ	Idelalisib	57%	6%	median 11 months	[206]
FL (R/R)	PI3Kα/δ	Duvelisib	42%	1%	n/a	[207]
FL (R/R)	PI3Kα/δ	Copanlisib	59%	14%	median 11.2 months	[195]
FL (R/R)	SYK	Fostamatinib	10%	0	median 4.6 months	[196]
FL (R/R)	SYK	Entospletinib	17%	0	median 5.7 months	[208]
FL (R/R)	SYK/JAK	Cerdulatinib	31%	15%	n/a	[199]
FL (R/R)	mTOR	Everolimus	61%	9%	n/a	[201]
MCL (R/R)	BTK	Ibrutinib	68%	21%	median 13.9 months	[209]
MCL (R/R)	BTK	Ibrutinib	78%	33%	n/a	[189]
MCL (R/R)	BTK	Tirabrutinib	92%	46%	median 11 months	[191]
MCL (R/R)	PI3Kδ	Idelalisib	40%	5%	median 3.7 months	[210]
MCL (R/R)	PI3Kα/δ	Copanlisib	64%	18%	n/a	[211]
MCL (R/R)	SYK	Fostamatinib	11%	0	median 3.8 months	[196]
MCL (R/R)	SYK	Entospletinib	18%	0	median 5.6 months	[208]
MCL (R/R)	SYK/JAK	Cerdulatinib	0	0	n/a	[199]
MCL (R/R)	mTOR	Temsirolimus	38%	3%	median 6.5 months	[212]
MCL (R/R)	mTOR	Temsirolimus	22%	0	median 4.8 months	[213]
MCL (R/R)	mTOR	Temsirolimus	40%	n/a	median 6.2 months	[214]
MZL (R/R)	BTK	Ibrutinib	48%	3%	median 14.2 months	[215]
MZL (R/R)	PI3Kδ	Idelalisib	47%	n/a	n/a	[193]
MZL (R/R)	PI3Kα/δ	Duvelisib	39%	6%	n/a	[207]
MZL (R/R)	PI3Kα/δ	Copanlisib	70%	9%	n/a	[195]
MZL (R/R)	SYK	Entospletinib	12%	0	median 5.5 months	[208]
WM (TN)	BTK	Ibrutinib	91%	0	69% at 24 months	[199]
WM (R/R)	BTK	Ibrutinib	75%	0	/	[189]
WM (R/R)	BTK	Tirabrutinib	94%	0	not reached	[216]
WM (TN)	BTK	Tirabrutinib	100%	0	not reached	[216]
WM/LPL(R/R)	PI3Kδ	Idelalisib	80%	n/a	n/a	[193]
WM/LPL(R/R)	SYK	Entospletinib	35%	0	median 10.9 months	[208]
WM/LPL(R/R)	PI3Kα/δ	Copanlisib	17%	0	n/a	[195]

* R/R, relapsed/refractory; TN, treatment naive; SLL, small lymphocytic lymphoma; n/a, not available.

**Table 2:** Common BCR-related features in different B cell malignancies.

Disease	Immunogenetic Characteristics	Activated BCR Pathway Components	Mechanism of BCR Pathway Activation
CLL	Biased IGHV gene repertoire Stereotyped BCRs	LYN, SYK, PI3K/AKT, BTK, PKCβ, NF-κB, NFAT, ERK	Reactivity with autoantigens Cell-autonomous BCR interactions
ABC DLBCL	Biased IGHV gene repertoire Stereotyped BCRs	SYK, PI3K/AKT, BTK, NF-κB	Reactivity with autoantigens Cell-autonomous BCR interactions CD79A/79B mutations Interaction with mutated MyD88
GCB DLBCL	Unbiased IGHV gene repertoire No stereotyped BCRs	LYN, SYK, PI3K/AKT	SHP1 deficiency (~50% of cases) Mannosylated BCRs (~40% of cases)
BL	Unbiased IGHV gene repertoire No stereotyped BCRs	LYN, SYK, PI3K/AKT	TCF3/ID3 mutations SHP1 deficiency
FL	Unbiased IGHV gene repertoire No stereotyped BCRs	LYN, SYK, PI3K/AKT (NF-κB in ~20% of cases)	Mannosylated BCRs (>80% of cases) SHP1 deficiency Reactivity with autoantigens (20–25% of cases)
MCL	Biased IGHV gene repertoire Stereotyped BCRs	LYN, SYK, PI3K/AKT, BTK, NF-κB	Reactivity with autoantigens (~70% of cases)
MZL	Biased IGHV gene repertoire Stereotyped BCRs	LYN, SYK, PI3K/AKT, NF-κB	Reactivity with autoantigens
WM	Biased IGHV gene repertoire Stereotyped BCRs	LYN, SYK, PI3K/AKT, BTK, NF-κB	Reactivity with autoantigens Interaction with mutated MyD88
